# Increased incidence of myelodysplastic syndrome and acute myeloid leukemia following breast cancer treatment with radiation alone or combined with chemotherapy: a registry cohort analysis 1990-2005

**DOI:** 10.1186/1471-2407-11-260

**Published:** 2011-06-21

**Authors:** Henry G Kaplan, Judith A Malmgren, Mary K Atwood

**Affiliations:** 1 Swedish Cancer Institute at Swedish Medical Center, Seattle, WA, USA; 2 HealthStat Consulting Inc., Seattle, WA, USA; 3 School of Public Health and Community Medicine, Department of Epidemiology, University of Washington, Seattle, WA, USA

## Abstract

**Background:**

Our objective was to measure myelodysplastic syndrome (MDS) and acute myelogenous leukemia (AML) risk associated with radiation and/or chemotherapy breast cancer (BC) treatment.

**Methods:**

Our study cohort was composed of BC patients diagnosed from 1990 to 2005 and followed up for blood disorders, mean length of follow up = 7.17 years, range 2-18 years. 5790 TNM stage 0-III patients treated with surgery alone, radiation and/or chemotherapy were included. Patients without surgery (n = 111), with stem cell transplantation (n = 98), unknown or non-standard chemotherapy regimens (n = 94), lost to follow up (n = 66) or 'cancer status unknown' (n = 67) were excluded. Rates observed at our community based cancer care institution were compared to SEER incidence data for rate ratio (RR) calculations.

**Results:**

17 cases of MDS/AML (10 MDS/7 AML) occurred during the follow up period, crude rate .29% (95% CI = .17, .47), SEER comparison RR = 3.94 (95% CI = 2.34, 6.15). The RR of MDS in patients age < 65 comparing our cohort incidence to SEER incidence data was 10.88 (95% CI = 3.84, 24.03) and the RR of AML in patients age < 65 was 5.32 (95% CI = 1.31, 14.04). No significant increased risk of MDS or AML was observed in women ≥ 65 or the surgery/chemotherapy-only group. A RR of 3.32 (95% CI = 1.42, 6.45) was observed in the surgery/radiation-only group and a RR of 6.32 (95% CI = 3.03, 11.45) in the surgery/radiation/chemotherapy group. 3 out of 10 MDS cases died of disease at an average 3.8 months post diagnosis and five of seven AML cases died at an average 9 months post diagnosis.

**Conclusions:**

An elevated rate of MDS and AML was observed among breast cancer patients < 65, those treated with radiation and those treated with radiation and chemotherapy compared to available population incidence data. Although a small number of patients are affected, leukemia risk associated with treatment and younger age is significant.

## Background

Breast cancer survival rates have improved over time with better chemotherapy and radiation treatment options and guidelines based on diagnostic tumor characteristics [1,2]. Leukemia after breast cancer treatment remains a concern for survivors but has been a rare event with incidence rates well documented in a variety of study settings [3-5]. Treatment-related translocation errors have been associated with the use of chemotherapy, especially alkylating agents and anthracyclines [6,7]. Current studies have linked secondary leukemia occurrence to older age [8-10], chemotherapeutic agents and dosage (epirubicin, doxorubicin and cyclophosphamide) [11,12], radiation treatment [13] and supportive treatment with granulocyte stimulating growth factor (GCSF) [14-16].

The peak incidence of leukemia post adjuvant chemotherapy has been reported most likely in the first 1-2 years post treatment with anthracyclines and 5-7 years with alkylating agents based on studies of chemotherapy related translocation errors in myelodysplastic syndrome (MDS) and acute myelogenous leukemia (AML) [17]. Current studies of radiation induced malignancies suggest a latency time frame of 1-5 years to occurrence of myeloid leukemia [18,19].

MDS first became a Surveillance, Epidemiology, and End Results (SEER) reportable cancer in 2001. A study by De Roos et al found 26% of MDS cases had previous cancer diagnoses treated with radiation with an associated increased risk of death [20]. Chemotherapy is not reported in the SEER Limited Use Data set, so analysis of chemotherapy or combination radiation/chemotherapy related to secondary MDS is not possible. A differential risk of leukemia related to chemotherapy, radiation, or both has been reported in studies of leukemia post treatment for Hodgkin's disease [21-23]. In the current study we report the incidence of MDS and AML in breast cancer patients treated with surgery alone, surgery plus radiation and/or chemotherapy and compare the rates to those obtained from SEER incidence data.

## Methods

A breast cancer registry of patients seen at our institution was created in 1990 containing detailed information on diagnosis, staging, surgery, chemotherapy, radiation therapy, tumor markers, and follow up status. Registry follow up is updated annually by a certified cancer registrar with information on recurrence, subsequent treatment and vital status, current through 2007. Vital and disease status information is obtained from chart review if the patient is still seen at the Institution, or through physician directed follow up letter if follow up care is provided elsewhere. Patients not under the care of a managing physician are contacted by mail using an IRB approved letter from their diagnosing physician requesting annual follow up information. If no response is received, the institution's cancer registry and the SEER 9 Seattle-Puget Sound Registry are reviewed for patient's vital and disease status [24]. Only IRB approved methods were used for patient follow up and data was input and stored in a password protected HIPAA compliant database. All analyses were conducted using de-identified data as per IRB and HIPAA guidelines. This project was reviewed and approved by the Institutional Review Board at our community based regional cancer center.

Patients who did not undergo surgery (n = 111), patients with incomplete chemotherapy records or non-standard treatment (n = 23+71 = 94), patients who underwent stem cell transplants (n = 98), were lost to follow up (n = 66) or had cancer status unknown at follow up (n = 67) were excluded. The analytic dataset included women who were treated for stage 0-III (AJCC 6) primary breast carcinoma and who had a minimum follow-up duration of 24 months [25]. The cohort of patients with a minimum follow up duration of two years was equal to 5,790.

A data review was done of the SEER 9 Seattle-Puget Sound Registry (SEER 9 S-PS) for all non-multiple myeloma bone marrow malignancies post breast cancer diagnosis of our registry patients to confirm complete ascertainment of MDS, AML or other leukemia occurrence. We included cases of MDS (histologic type ICD-O-3 codes 9980, 9982-9987, 9989), AML (histologic type ICD-O-3 codes 9840, 9861, 9866-9867, 9871-9874, 9895-9897, 9910, 9920), chronic lymphocytic leukemia (CLL) (histologic type ICD-O-3 codes 9823), chronic myelogenous leukemia (CML) (histologic type ICD-O-3 codes 9863, 9875, 9876, 9945, 9946) and acute lymphocytic leukemia (ALL) (histologic type ICD-O-3 codes 9826, 9835-9837) [26]. Cases previously identified as refractory anemia with excess blasts (RAEB) were coded as MDS 9983.

Crude cumulative incidence rates were calculated for the two and five year follow-up cohorts. Chemotherapy regimens were reviewed and classified as either anthracyline-containing or non-anthracycline-containing regimens for the purpose of this analysis. For time to leukemia diagnosis, observation time began on the breast cancer diagnosis date and censoring occurred on the leukemia diagnosis date, date of death or date of last follow-up. Time from treatment to leukemia diagnosis date was recalculated for two surgery only patients subsequently treated with chemotherapy and radiation for recurrence.

Expected age adjusted incidence rates per 100000 person years were calculated using SEERstat version 6.5.2 software (NCI) with cases identified from SEER 9 S-PS for years 2001-2005 for MDS and 1990-2006 for AML, CML, ALL, and CLL [24]. MDS cases were identified using ICD-O-3 codes matched to the SEERstat recode ICD-O-3 list [26,27]. Observed age adjusted incidence rates by person-years were calculated using the number of events divided by the total number of person years of follow-up with exact 95% confidence intervals obtained from the Poisson distribution [28]. Cumulative 15 year incidence rates of MDS/AML were calculated with adjustment for survival using Kaplan-Meier plots [29]. Ninety-five percent confidence intervals (CIs) for cumulative incidence were calculated using the bootstrap method; use of arcsine and logarithmic methods yielded highly similar results [30]. Cox regression analysis was used to assess the relationship between age and treatment and time to MDS/AML incidence [29]. Interval from breast cancer diagnosis to MDS/AML incidence by treatment groups was calculated using the Kaplan Meier method [29].

## Results

Average patient age was 57 years, range 21-94 years and average follow up for patients alive at follow up was 7.16 years, range 2-18 years. 69% (n = 4,014) of the cohort were less than age 65 and 31% (n = 1,776) were 65 or older (table 1). The total number of person years analyzed was 40,650, with patients diagnosed in 1998 contributing the most person years. We included TNM stage 0-III with 13% stage 0, 45% stage I, 35% stage II, and 7% stage III. 420 patients were treated with surgery only (7%), 2764 had surgery/radiation therapy (48%), 640 had surgery/chemotherapy (11%) and 1966 had combined surgery/radiation/chemotherapy treatment (34%). Treatment received differed significantly by age < 65 and ≥ 65 (chi square = 920.17, p < .001). Surgery only and surgery/radiation were roughly equivalent between the two age groups but only 8% of the surgery/chemotherapy group was 65 and older and 12% of the surgery/radiation/chemotherapy group was 65 or older. Eighty percent of chemotherapy patients received one of four standard treatments: 1) anthracycline and cyclophosphamide (AC) (19%), 2) cyclophosphamide, anthracycline and fluorouracil (CAF) (13%), 3) cyclophosphamide, methotrexate and fluorouracil (CMF) (22%), and 4) AC with a taxane (26%). 76% of chemotherapy patients received anthracyclines (n = 1,973). Nine hundred seventy two of 2,081 chemotherapy patients (46.7%) received G-CSF in varying amounts during chemotherapy treatment.

**Table 1:** Descriptive Statistics (n = 5790)

Variable	
Age: mean (range)	57.40 (21-94)

TNM Stage:	N (%)

0	736 (12.7%)

I	2625 (45.3%)

IIa	1298 (22.4%)

IIb	729 (12.6%)

IIIa	239 (4.1%)

IIIb	116 (2.0%)

IIIc	47 (.8%)

Estrogen receptor positive	4268 (73.7%)

Progesterone receptor positive	3628 (62.7%)

Treatment:	N (%)

Surgery only	420 (7.3%)

Surgery/radiation	2764 (47.7%)

Surgery/chemotherapy	640 (11.1%)

Surgery/radiation/chemotherapy	1966 (34.0%)

Anthracycline regimen	1973 (75.7%)

Non-anthracycline regimen	646 (24.3%)

Tamoxifen	3554 (61.4%)

Follow up years: mean (range)	7.02 (.20, 18.15)

Vital Status:	N (%)

Alive, NED*	4741 (81.9%)

Alive with this cancer	123 (2.1%)

Alive with other cancer	62 (1.1%)

Expired, NED	354 (6.1%)

Expired with this cancer	388 (6.7%)

Expired with other cancer	121 (2.1%)

Expired, treatment complications	1 (.0%)

*NED: no evidence of disease

There were 17 incident cases of MDS/AML with ten MDS cases (7 with initial treatment only/no treatment for recurrence and three cases with initial treatment/treatment for recurrence) and 7 AML cases (5 cases initial treatment/no treatment for recurrence and two treatment related AML initially diagnosed as MDS progressing to AML, one of which was treated for recurrence). The median age at breast cancer diagnosis of the MDS/AML patients was 57 years (range 33-76 years) with no significant difference in distribution of MDS/AML incidence by age. Two patients had surgery only for initial treatment with subsequent radiation and chemotherapy for recurrent disease, seven had surgery/radiation, one had surgery/chemotherapy and seven had surgery/radiation/chemotherapy treatment. The two surgery only patients were < 65, the surgery/chemotherapy patient was ≥ 65, two of the 7 surgery/radiation patients were < 65 and six of the 7 surgery/radiation/chemotherapy patients were < 65 (chi square = 7.56, p = .056). Of the 16 MDS/AML patients receiving radiation therapy all were standard dose of either 5000-5580 centi-Gray units (cGy) with a 1000-1440 cGy boost or 4500-4680 cGy with a 1400 cGy boost delivered at a number of different local radiation facilities. Two patients were administered an additional 4860-5040 cGy to the lymph nodes.

**Figure 1:**
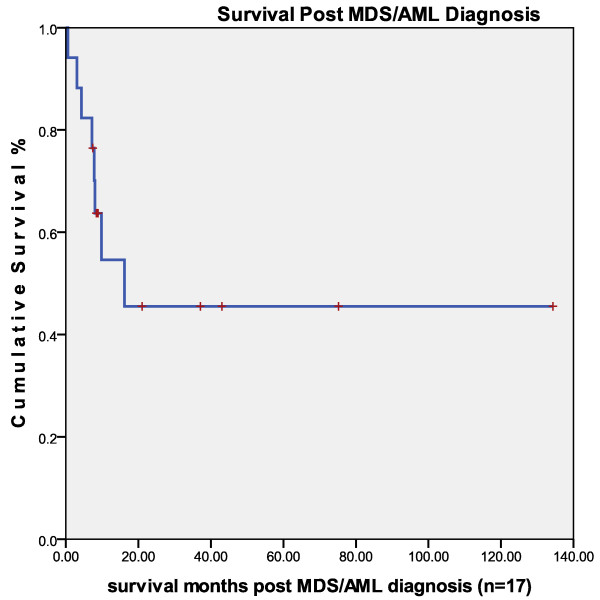
**Survival time in months post myelodysplastic syndrome/acute myelogenous leukemia diagnosis after treatment for primary breast cancer (n = 17)**.

Median survival of MDS/AML patients post diagnosis was 9 months, range .5 to 134 months. Median survival for the seven 'alive at follow up' MDS patients was 21 months, range 8-75 months. Three of the 10 MDS post breast cancer patients died of MDS at an average of 3.8 months post MDS diagnosis, range .5-8 months and five of the seven AML post breast cancer patients died of AML at a median of 8 months post AML diagnosis (Figure 1).

Cytogenetic reports were available on five of 17 MDS/AML patients. One patient demonstrated an 11q23 translocation, consistent with anthracycline induced acute leukemia [6]. Another patient had chromosome 5 and 7 abnormalities consistent with alkylating agent-induced acute leukemia, as well as de novo leukemia and MDS [31].

Median time from breast cancer diagnosis to MDS/AML diagnosis was 4.35 years, range .67-13.6 years. Median time from treatment with radiation or chemotherapy was 3.06 years, range = .41-12.95 years. The latter number takes into account the chemotherapy and radiation treatment start dates for the two surgery only patients that had recurrences and the treatment start date instead of the breast cancer diagnosis date for the other 15 patients. A paired samples t-test comparison of mean time to leukemia incidence post initial or first recurrence treatment for the two surgery only initial treatment patients was not significant.

In our cohort, two patients developed CML, two ALL, and five CLL (n = 9) (table 2). Average age at diagnosis was 73 years, range 53 to 88 years. The CLL, ALL and one of the two CML patients were diagnosed with leukemia at ≥ 65 years of age. Eight out of nine received surgery/radiation treatment, one had surgery only and none had chemotherapy as initial treatment. Average time from breast cancer diagnosis to CML, ALL, or CLL diagnosis was 4.92 years, range .25 to 8.92 years.

**Table 2:** CML/CLL/ALL Case Description (n = 9)

Age at dx	Disease stage at dx	Chemotherapy	Radiation	Leukemia Type	Time from breast dx to leukemia (months)	Time from leukemia dx to last fu (months)
79	I	none	yes	CLL	37	57 (Alive)

64	I	none	yes	CLL	61	56 (Dec.)

74	I	none	yes	CLL	96	20 (Alive)

61	II	None	no	CLL	79	109 (Alive)

58	I	None	yes	CLL	110	70 (Alive)

50	I	recurrence 1993	yes	CML	33	9 (Dec.)

73*	I	None	yes	CML	3	14 (Dec.)

76	II	None	yes	ALL	38	19 (Dec.)

81	I	None	yes	ALL	81	4 (Dec.)

* Cytogenetics: Philadelphia chromosome resulting from a 9;22 translocation designated as 46, XX, t(9;22)(q34;q11 2).

**Figure 2:**
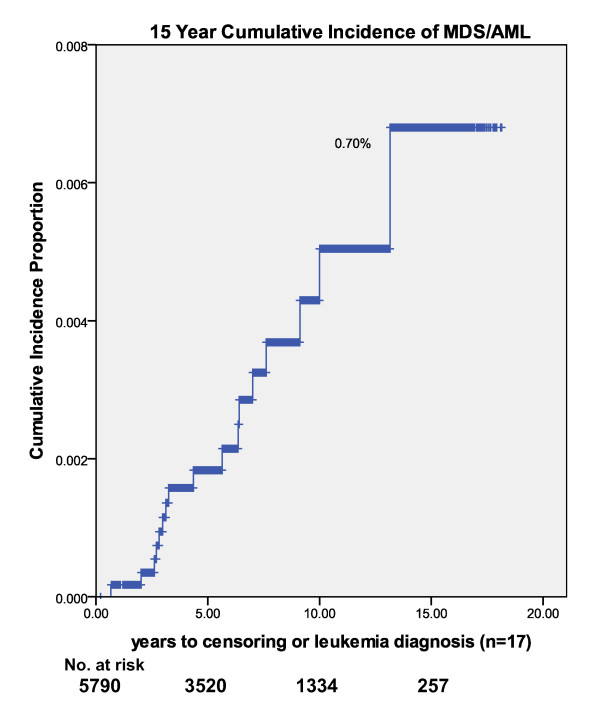
**15 year cumulative incidence of myelodysplastic syndrome/acute myelogenous leukemia (n = 17)**.

We calculated crude incidence rates of MDS/AML and all leukemia types (MDS, AML, CML, CLL, ALL) for two and five year minimum follow up by treatment combinations (tables 3 and 4). Crude rate of MDS/AML incidence was .29% (95% CI = .17, .47). By treatment category with minimum 2 years follow up the crude rates were .25 (7/2764) surgery/radiation (95% CI = .10, .52), .16 (1/639) surgery/chemotherapy (95% CI = .00, .87) and .46 (9/1959) surgery/radiation/chemotherapy (95% CI = .21, .87). No difference in MDS/AML was observed in the anthracycline compared to the non-anthracycline adjuvant chemotherapy treatment group. Crude all leukemia incidence rate (MDS, AML, CML, CLL, ALL) was .45% for two year minimum follow up. Crude rates of MDS/AML or combined all leukemia did not differ significantly by treatment regimen. Using Kaplan-Meier one-minus-survival curve estimates, the 5-year MDS/AML cumulative incidence rate was .20%, the 10-year rate was .50% and the 15-year rate was .70% (Figure 2).

**Table 3:** Crude MDS/AML rates by treatment category

Treatment category	% (cases/no. in category) (95% CI)
2 years of follow up (n = 5790)	

All patients	.29 (17/5790) (.17, .47)

Surgery only	0 (0/420) (0)

Surgery/radiation	.25 (7/2764) (.10, .52)

Surgery/chemotherapy	.16 (1/639) (.00, .87)

Surgery/radiation/chemotherapy	.41 (9/1959) (.21, .87)

Anthracycline regimen	.25 (5/1968) (.08, .59)

Non-anthracycline regimen	.47 (3/633) (.10, 1.38)

5 years of follow up (n = 4179)	

All patients	.41 (17/4179) (.24, .65)

Surgery only	0 (0/222) (0)

Surgery/radiation	.24 (5/2058) (.08, .57)

Surgery/chemotherapy	.22 (1/461) (.01, 1.20)

Surgery/radiation/chemotherapy	.42 (6/1440) (.15, .90)

Anthracycline regimen	.15 (2/1368) (.02, .53)

Non-anthracycline regimen	.56 (3/531) (.12, 1.64)

**Table 4:** Crude all leukemia rates by initial treatment category (MDS, AML, CML, CLL, ALL)

Treatment category	% (cases/no. in category) (95% CI)
2 years of follow up (n = 5790)	

All patients	.45 (26/5790) (.29, .66)

Surgery only	.24 (1/418) (.01, 1.33)

Surgery/radiation	.54 (15/2764) (.30, .89)

Surgery/chemotherapy	.16 (1/640) (.00, .87)

Surgery/radiation/chemotherapy	.46 (9/1968) (.21, 1.64)

5 years of follow up (n = 4179)	

All patients	.50 (21/4179) (.31, .77)

Surgery only	.45 (1/220) (.01, 2.51)

Surgery/radiation	.63 (13/2058) (.34, 1.08)

Surgery/chemotherapy	.22 (1/461) (.01, 1.20)

Surgery/radiation/chemotherapy	.42 (6/1440) (.15, .90)

Ten cases of MDS occurred during the follow up period compared to 2.72 expected from SEER 9 S-PS age adjusted rates. The SEER expected MDS incidence rate for women < 65 years was 1.6/100000 and our observed rate was 17.5/100000, RR = 10.88 (95% CI = 3.84, 24.03) (table 5). The SEER expected MDS incidence rate for women ≥ 65 years was 31.6/100000 and the observed rate was 41.6/100000, RR = 1.31 (95% CI = 0.47, 2.84). The SEER expected AML incidence rate for women < 65 years was 2.0/100000 and the observed rate was 10.5/100000, RR = 5.32 (95% CI = 1.32, 14.04). The SEER expected AML incidence rate for women ≥ 65 years was 14.0/100000 and the observed rate was 33.3/100000, RR = 2.38 (95% CI = 0.73, 5.61). Overall, 17 cases of MDS/AML (10 MDS/7 AML) occurred during the follow up period compared with 4.29 expected based on SEER rates (RR = 3.94, 95% CI = 2.34, 6.15).

**Table 5:** Expected and observed incidence rates of MDS/AML by age per 100000 person years.

Leukemia Type	Age	Expected Rate*	Person years	Observed Rate	Expected Cases	Observed Cases	Rate Ratio (95% CI)
MDS	< 65	1.6	28538	17.5	0.46	5	10.88 (3.84, 24.03)

	≥ 65	31.6	12027	41.6	3.80	5	1.31 (0.47, 2.84)

	All	6.7	40565	24.7	2.71	10	3.70 (1.85, 6.54)

AML	< 65	2.0	28538	10.5	0.57	3	5.32 (1.31, 14.04)

	≥ 65	14.0	12027	33.3	1.68	4	2.38 (0.73, 5.61)

	All	4.0	40565	17.3	1.62	7	4.32 (1.85, 8.45)

MDS/AML	< 65	3.6	28538	28.1	1.03	8	7.82 (3.54, 14.77)

	≥ 65	45.6	12027	74.8	5.48	9	1.64 (0.79, 2.98)

	All	10.6	40565	41.9	4.30	17	3.94 (2.34, 6.15)

*SEER 9 incidence rates, Seattle-Puget Sound Region [24]

**Figure 3:**
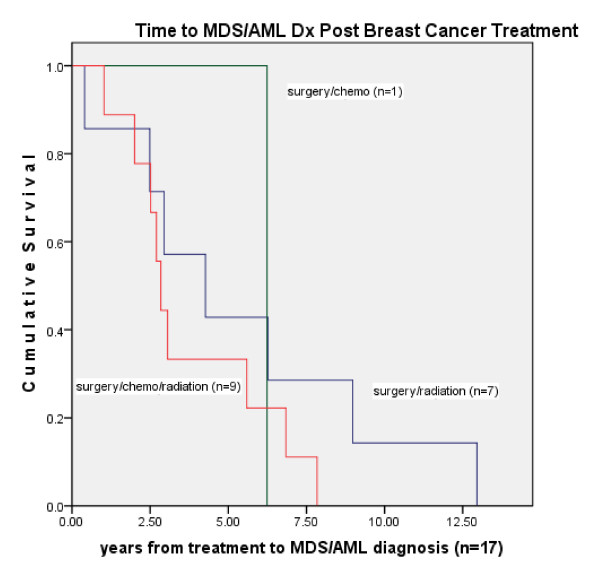
**Time in years from categorical breast cancer treatment to diagnosis of myelodysplastic syndrome or acute myelogenous leukemia (n = 17)**.

Comparing the rate of MDS/AML in our cohort by treatment category to the SEER 9 S-PS region MDS/AML incidence rates, the rate ratio among patients treated with surgery/radiation (n = 7) was 3.32 (95% CI = 1.42, 6.45) and surgery/radiation/chemotherapy (n = 9) was 6.32 (95% CI = 3.03, 11.45). No cases of MDS/AML were observed in the surgery only group and one case occurred in the surgery/chemotherapy group (RR = 1.88, 95% CI = .11, 8.30) (table 6). MDS/AML incidence distribution was not significantly different by age but treatment received was significantly different by age with younger patients receiving more chemotherapy. In a Cox regression model measuring time to leukemia diagnosis in months and correcting for age, initial treatment was not significant in the model (n = 5,790), (-2 log likelihood = 270.52, chi square = 3.65, 2 degrees of freedom, p = .456). Due to zero cases of MDS/AML in the surgery only group when the groups including treatment for recurrence were used, a second model could not be run. In survival analysis the four treatment groups did not differ significantly from time of initial breast cancer diagnosis to time of MDS/AML diagnosis (log rank test = 4.327, p = .226) with the majority of the surgery/radiation/chemotherapy group MDS/AML incidence occurring within 5 years of treatment (n = 7) (Figure 3). There was no significant difference in leukemia incidence by GCSF treatment with 972/2081 chemotherapy patients treated with GCSF, Pearson chi square = .877, p = .349.

**Table 6:** Expected and observed incidence rates of MDS/AML by treatment group per 100,000 person years.

	Expected Rate*	Person years	Observed Rate	Expected Cases	Observed Cases	Rate Ratio (95% CI)
SEER 9 S-PS	10.6	----------	----------	----------	----------	----------

Surgery only	10.6	2525	0	.27	0	0

Surgery/ radiation	10.6	19776	35.4	2.10	7	3.32 (1.42, 6.45)

Surgery/ chemotherapy	10.6	4985	20	.53	1	1.88 (0.11, 8.30)

Surgery/ radiation/ chemotherapy	10.6	13365	67.3	1.42	9	6.32 (3.03, 11.45)

All treatment categories	10.6	40565	41.9	4.30	17	3.94 (2.34, 6.15)

*The SEER 9 incidence rate age 20-80+ for MDS/AML was used for the comparison rate for all treatment categories, Seattle-Puget Sound Region [24]

## Discussion

We found a statistically significant increased risk of MDS and AML among post treatment breast cancer patients (rate ratio = 3.95, range 2.34-6.15). Although the increase in risk is significantly higher than expected in the general population when we compare our rates to those for the same age and gender using SEER data, the rate represents a small actual number of affected patients (17/5790, .29%) Three of the ten MDS patients (30%) and 5 of the 7 AML patients (71%) died of disease. The risk of MDS in women < 65 years of age was 11 times higher than SEER 9 S-PS estimates and risk of AML in women < 65 was 5 times higher. While the incidence of AML/MDS was substantial among women aged 65 and over, it was not statistically significant. This is perhaps due to a higher background incidence rate of MDS/AML in women 65 and older. A three-fold increase in risk was observed among patients treated with radiation alone and a six fold increase in risk among patients treated with radiation and chemotherapy indicating a possible synergistic effect of radiation and chemotherapy.

There was no increased risk of MDS/AML in the surgery/chemotherapy group or the surgery only group. Both of these groups were small in number and represented a small percentage of the total cohort, treatment with surgery only at 7% (n = 420) and surgery/chemotherapy at 11% (n = 640). With a limited number of chemotherapy only patients even with many years of follow-up it is unlikely we would be able to ascertain a small increased risk if it is present.

The strengths of our study are the following: complete case ascertainment with follow up from our registry and linkage to the Seattle-Puget Sound SEER database, complete treatment information on each patient, inclusion of MDS diagnosis, a large cohort with substantial follow up years post treatment, and a relatively large number of index patients. In our previous report on leukemia post breast cancer a comparison group with specific incidence of MDS and AML was not as yet available from SEER data. In our current analysis we report the treatment specific crude rates and rate ratios using the SEER population data for comparison. We have also moved the two patients treated initially with surgery only into the appropriate post recurrence treatment category, leaving the surgery only group without any cases. A larger cohort or perhaps longer follow up such as we have now may be necessary to characterize rates of treatment related MDS/AML using a population based comparison group [5]. We did not observe an association of increased risk with anthracycline therapy previously or in the current study. We did not find an association with GCSF treatment, despite the fact that this possibility has been suggested in other studies [14].

A limitation of our study is the absence of cytogenetic studies on all patients which if available can confirm treatment related leukemia. Time proximity from primary breast cancer to MDS/AML occurrence may indicate treatment related disease. The two cases with questionable time from treatment to leukemia incidence were one AML patient with less than 1 year post treatment and one patient with greater than 10 years post treatment both of whom were over 75 years of age. A very short (< 1 year) or very long latency period (>10 years) post treatment may indicate non-treatment related disease especially in an older adult. Our comparison of leukemia incidence post treatment by chemotherapy regimen received may lack power due to most patients (80%) receiving an anthracycline based therapy and a much smaller number receiving non-anthracyline chemotherapy. This is the first study measuring MDS and comparing incidence of MDS post breast cancer treatment to nationally reported rates. A drawback of this comparison may be underreporting of MDS as it is a newly reportable disease which may inflate the risk ratio comparison. Although this is a possibility we sought to reduce the chance by using rates from the same area in which the community based cancer treatment center is located.

Martin et al in their SEER data analysis of AML in breast cancer survivors found an increased risk of AML in younger women with stage III compared to stage I disease (< 50 and 50-64 years of age) but not in older women (65 and older) as we have in the present study [32]. Among the AML cases (n = 7), 3 had a stage III diagnosis, age 63, 64, and 65 years. All MDS cases in our cohort (n = 10) occurred in women diagnosed with stage 0-II breast cancer. Martin et al found risk of post treatment AML to be associated with age younger than 65 which has also been reported in a National Cancer Institute study of new malignancies following breast cancer [32,33]. The NCI study group reported an increased risk of acute non-lymphocytic leukemia in younger vs. older women with overall peak risk for all ages occurring 1-4 years post diagnosis of breast cancer [33]. Risk was highest among women less than 40 years of age and increased risk continued until >10 years of follow up with no increased risk at ages greater than 70 years.

An increased risk of leukemia associated with combination radiation and chemotherapy treatment has been reported from randomized clinical trials (RCTs) with comprehensive follow up of study participants for leukemia post breast cancer treatment [4,11,12,34-37]. Renella et al evaluated the risk of AML only post breast cancer treatment among a cohort of 6360 patients diagnosed between 1970 and 1999 and followed to 2000 finding a cumulative risk of AML equal to 0.20%, a 3.5-fold increased risk over the general population [38]. Risk was significantly increased among women greater than 70 years of age and those treated with radiotherapy. In a case-control study within a cohort an increased risk of acute non-lymphocytic leukemia was found among patients treated with regional radiotherapy alone, alkylating agents alone and combined radiation and chemotherapy with the highest risk ratio among radiation/chemotherapy patients [3]. The effect of radiation is not well characterized from summaries of study results as most observations are drawn from randomized clinical trials that do not include radiotherapy only patients and do not include identification of MDS.

We did observe a number of cases of ALL, CML, and CLL in our patient population post breast cancer treatment. All but one of these patients received radiation therapy for their breast cancers. Each of these diseases has been associated with exposure to ionizing radiation, although there has been some uncertainty with regard to CLL [39-41]. However, the number of cases in our cohort is too small to allow us to draw any additional conclusions.

Only a few cohort studies have been conducted which include radiation treatment patients as a separate group [3,38]. In a review of the epidemiologic literature, ionizing radiation exposure has been linked to cancer with leukemia being particularly sensitive to induction especially at younger ages [42]. Radiation can interact with other carcinogens such as chemotherapeutic agents and host factors such as age. Therefore combination therapy may confer a higher risk of secondary leukemia in younger vs. older women which may offer another explanation for our observed absence of increased risk in older women. The increased risk among women less than age 65 treated with radiation suggests a possible role of radiation in the pathogenesis of treatment related AML and MDS. Previously reported leukemic effects of chemotherapy may have been more compelling with daily oral dosing and higher cumulative doses of cyclophosphamide given in the past, as well as the use of agents such as melphalan, which appears to be more leukemogenic than other alkylating agents [3,36]. Current intermittent drug administration at lower cumulative doses may not be as leukemogenic as past treatment regimens and we did not observe any increased risk in the smaller group of chemotherapy only patients. The finding of a higher risk associated with radiation and chemotherapy combination therapy as opposed to either modality alone has also been reported in the treatment of Hodgkin's disease [23].

## Conclusions

Our findings suggest an increased risk of MDS/AML with radiation treatment in this patient population. An increased risk related to radiation therapy raises a number of issues. First, it would add another dimension to the question of whether a woman might choose a mastectomy as opposed to lumpectomy followed by radiation, especially if she is to receive adjuvant chemotherapy. Second, it raises the question of whether or not differential radiation exposure from partial breast irradiation techniques as opposed to more traditional techniques might change the radiation-induced risk of MDS/AML.

Current diagnosis and treatment patterns include more early stage disease and an increase in the number and percent of patients treated with radiation only post surgery, especially among younger women [43]. Recent studies suggest that carcinogenesis related to radiation exposure may have different mechanisms at different ages, leaving younger women more susceptible to tumor promotion than older women [44]. An increased risk of treatment related leukemia in younger age groups more susceptible to the promotion effect of radiation adds urgency to the exploration of factors which differentiate DCIS and early stage invasive breast cancer cases that require aggressive therapy from those that do not [45-47]. Although the absolute number of MDS/AML post breast cancer treatment is small, the risk of post treatment leukemia must be considered in the choice of therapy for breast cancer patients. Future studies of MDS and AML incidence post radiation and chemotherapy are needed to fully inform patients, survivors and physicians of treatment risk.

## Abbreviations

BC: breast cancer; MDS: myelodysplastic syndrome; AML: acute myelogenous leukemia; CML: chronic myelogenous leukemia; CLL: chronic lymphocytic leukemia; ALL: acute lymphocytic leukemia; RAEB: refractory anemia with excess blasts; ICD-O-3: International Classification of Disease for Oncology-3; NCI: National Cancer Institute; DCIS: ductal carcinoma in situ; SEER: Surveillance, Epidemiology, and End Results; TNM: tumor, node and metastasis; NED: no evidence of disease; RR: rate ratio; cGY: centi-Gray units; RCT: randomized clinical trial; dx: diagnosis; Dec.: deceased; fu: follow up; No.: number.

## Competing interests

The authors declare that they have no competing interests.

## Authors' contributions

HK, JM, and MA made substantial contributions to the conception and design, acquisition of data, or analysis and interpretation of the data and have been involved in drafting the manuscript or revising it critically. JM conducted the statistical analyses with review by HK. All authors read and approved the final manuscript.

## Pre-publication history

The pre-publication history for this paper can be accessed here:

http://www.biomedcentral.com/1471-2407/11/260/prepub
